# Measurement of multiple cytokines for discrimination and risk stratification in patients with Chagas’ disease and idiopathic dilated cardiomyopathy

**DOI:** 10.1371/journal.pntd.0008906

**Published:** 2021-03-23

**Authors:** Yong Wang, Niels Wessel, Franziska Kohse, Adnan Khan, Heinz-Peter Schultheiss, Maria da Consolaҫão V. Moreira, Thomas Walther

**Affiliations:** 1 Department of Cardiology and Angiology, Hannover Medical School, Hannover, Germany; 2 Department of Physics, Humboldt-Universität zu Berlin, Berlin, Germany; 3 Institute of Medical Biochemistry and Molecular Biology, University Medicine Greifswald, Greifswald, Germany; 4 Department of Cardiology, Charité, Campus Benjamin Franklin, Berlin, Germany; 5 Department of Internal Medicine, UFMG, Belo Horizonte, Brazil; 6 Department of Pharmacology and Therapeutics, School of Medicine and School of Pharmacy, University College Cork, Cork, Ireland; Universidade Federal de Minas Gerais, BRAZIL

## Abstract

Chagas’ disease (CD), caused by the hemoflagellate protozoan, *Trypanosoma cruzi*, is endemic in most countries of Latin America. Heart failure (HF) is often a late manifestation of chronic CD, and is associated with high morbidity and mortality. Inflammatory processes mediated by cytokines play a key role in the pathogenesis and progression of CD. Keeping in view the inflammatory nature of CD, this study investigated the possible role of 21 different inflammatory cytokines as biomarkers for prediction and prognosis of CD. The plasma concentration of these cytokines was measured in a group of patients with CD (n = 94), and then compared with those measured in patients with dilated cardiomyopathy (DCM) from idiopathic causes (n = 48), and with control subjects (n = 25). Monovariately, plasma levels of cytokines such as stem cell growth factor beta (SCGF beta), hepatocyte growth factor (HGF), monokine induced by interferon gamma (CXCL9), and macrophage inhibitory factor (MIF) were significantly increased in CD patients with advanced HF compared to control group. None of the cytokines could demonstrate any prognostic potency in CD patients, and only MIF and stromal derived factor-1 alpha (CXCL12) showed significance in predicting mortality and necessity for heart transplant in DCM patients. However, multivariate analysis prognosticated a large proportion of CD and DCM patients. In CD patients, HGF and Interleukin-12p40 (IL-12p40) together separated 81.9% of 3-year survivors from the deceased, while in DCM patients, CXCL12, stem cell factor (SCF), and CXCL9 together discriminated 77.1% of survivors from the deceased. The significant increase in plasma concentrations of cytokines such as HGF and CXCL9 in CD patients, and the ability of these cytokines to prognosticate a large proportion of CD and DCM patients multivariately, encourages further studies to clarify the diagnostic and prognostic potential of cytokines in such patients.

## Author summary

Chagas’ disease (CD), caused by the parasite, *Trypanosoma cruzi*, is endemic in most of Latin America. Cardiac involvement is the most frequent in chronic CD. Heart failure (HF) is often a late manifestation of chronic CD and is associated with high morbidity and mortality. Early identification of patients with CD would be desirable as early intervention may help improve prognosis. Inflammatory biomarkers can play a vital role in early diagnosis, as inflammation mediated by cytokines plays an important role in pathogenesis and progression of CD.

This study investigated the possible role of 21 different inflammatory cytokines as biomarkers for discrimination and risk stratification of CD and idiopathic dilated cardiomyopathy (DCM). Plasma concentrations of various cytokines were significantly increased in CD and DCM patients with advanced HF, however, they were unable to show predictive or prognostic potency in CD. Using various cytokines together, analyses prognosticated fatal outcome in a large proportion of CD and DCM patients, but could still not discriminate CD from idiopathic DCM.

Our promising data encourages research on identifying further inflammatory biomarkers that could, in combination with the 21 measured in this study, be used as tools for early diagnosis and prognosis of CD.

## Introduction

Chagas’ disease (CD) (also known as American trypanosomiasis) is a parasitic disease that is endemic throughout most of Central and South America. It is caused by the hemoflagellate protozoan, *Trypanosoma cruzi* (*T*. *cruzi*), which is transmitted to humans and other mammals mostly by an insect vector, the blood sucking bugs of the subfamily Triatominae (family Reduviidae) [1]. Regarded by World Health Organization (WHO) as one of the thirteen most neglected tropical diseases in the world [2], CD continues to be a major source of financial, social and health burden to the affected individuals and countries [3]. In addition, the influx of immigrants from countries endemic for disease in recent times has meant that CD is quickly becoming a major health concern in North America, in many parts of Europe, and the Western Pacific, where an increasing number of infected individuals have been identified [4,5]. Nearly 10 million people are infected worldwide, and more than 25 million people are at risk of the disease [6].

Human exposure to *T*. *cruzi* may lead to an acute Chagas’ infection, which usually lasts for 4–8 weeks. The chronic phase that follows persists for lifespan of the host [7]. The typical clinical manifestations of chronic phase are related to pathological involvement of heart, esophagus, colon, or a combination, and are grouped into three major forms: cardiac, digestive, and cardio-digestive [8]. The cardiac form is the most frequent and serious manifestation of chronic CD. It develops in 20–30% of individuals and usually leads to abnormalities of the conduction system, arrhythmias, apical aneurysms, thromboembolism, heart failure (HF), and sudden death [9].

HF is characterized by the activation of various inflammatory processes that result in increased levels of inflammatory markers such as interleukin-6 (IL-6) and C-reactive protein (CRP) [10]. This activation may occur due to several reasons such as myocardial damage, reduced cardiac output, and/or hemodynamic overload. A vicious circle is present between HF and inflammation as inflammatory markers have been found to be associated with worsening of cardiac function and poor prognosis [11].

Cytokines belong to a group of relatively low molecular weight, pharmacologically active proteins, which regulate host responses to infection, immune responses, inflammation, and trauma [12]. Some of the important pro-inflammatory cytokines implicated in the progression of HF are tumour necrosis factor (TNF) (formerly named TNF alpha) and IL-1 [13]. These cytokines are secreted by all nucleated cell types present in the myocardium, including cardiac myocytes. The “cytokine hypothesis” for HF states that HF progresses, at least partially, due to the toxic effects of the endogenous proinflammatory cytokine cascades on the heart and the peripheral circulation [14]. It emphasizes on the point that cytokines may not be the initial cause of HF, but rather that the over expression of proinflammatory cytokines contributes to the disease progression of HF. Thus, the expression of cytokines may represent a biological mechanism that is responsible for worsening HF.

In chronic CD patients with cardiomyopathy, there is progressive and persistent inflammation of myocardial fibers that leads to gradual impairment of contractile function and dilatation of all four chambers of the heart [13]. With time, the myocytes are gradually lost due to inflammatory tissue destruction, and the dead myocytes are then replaced by intense fibrosis, which predispose the patient to development of HF, ventricular arrhythmias, and other serious pathologies [15].

Keeping in view the inflammatory nature of CD, this study investigated the possible role of serum cytokines as biomarkers for diagnosis and prognosis of CD. In our earlier studies, we used monovariate analysis to investigate the diagnostic and prognostic potentials of three cytokines, hepatocyte growth factor (HGF), stem cell growth factor beta (SCGF beta), and monokine induced by interferon gamma (CXCL9; also known as MIG) in CD and idiopathic dilated cardiomyopathy (DCM) [16–18]. Here, we quantified additional 18 cytokines and tested whether multivariate analyses would better diagnose and prognosticate patients with CD and patients with DCM than single cytokines.

Our aims were: (1) to measure plasma concentrations of 21 different cytokines in CD and DCM patients and compare it with those in healthy human volunteers and to investigate a possible correlation with the functional New York Heart Association (NYHA) class and left ventricular ejection fraction (LVEF); (2) to compare the circulating levels of each cytokine in CD patients with those having DCM, characterizing them as possible diagnostic markers to differentiate between the two types of HF; (3) to evaluate the prognostic value of plasma cytokines for mortality and the necessity for heart transplant in CD and DCM; and (4) to test whether a fingerprint of cytokines identified by multivariate analyses can improve the diagnostic and predictive capacity of cytokines.

## Methods

### Ethics statement

The study was approved by the institutional review committee, and all patients gave written consent. The study population consisted of a prospective cohort of 142 adult human subjects from the Heart Failure Center of the Felicio-Rocho Hospital, Brazil, enrolled between July 2001 and January 2005. Ninety-four (94) consecutively recruited patients with at least two positive serologies for CD (Chagas group [CD]) and forty-eight (48) patients with negative serology for CD (idiopathic DCM group) were studied and compared with twenty-five (25) adjusted gender- and age-matched healthy subjects.

All clinical data was obtained by the same investigator as reported previously [19–21], and included the medical history, physical examination, and resting electrocardiogram. M-mode, two-dimensional, and Doppler echocardiographic measurements were performed with an ultrasonographic system (Sequoia C256, Acuson, AcusonInc, Mountain View, CA, USA) according to the recommendations of the American Society of Echocardiography. Left ventricular systolic dysfunction was defined by a left ventricular ejection fraction (LVEF) of < 50% as assessed by transthoracic echocardiography (Teichholz method).

Other structural cardiac diseases and co-morbidities were excluded by medical history, physical examination, electrocardiogram, M-mode, two-dimensional, and Doppler echocardiography, or coronary arteriography: valvular heart disease, coronary artery disease, congenital disease, acute myocarditis, hypertensive disease, renal failure (plasma creatinine > 0.2 mmol/L), chronic pulmonary disease, hepatic cirrhosis, active infections, and endocrine disease. Patients with ventricular dysfunction received standard pharmacologic therapy according to the New York Heart Association (NYHA) functional class and were clinically stable for at least 30 days.

Patients with advanced refractory HF were considered as heart transplant candidates and listed for the procedure in the absence of any contraindication. The control group was composed of healthy subjects attending the hospital clinics for a physical check-up. They had no cardiac symptoms or history, no co-morbidities, and took no medication.

The study patients were prospectively defined and subdivided into 5 groups: Group 1 (n = 46), CD without ventricular systolic dysfunction (LVEF > 50%); Group 2 (n = 25), CD with ventricular systolic dysfunction (LVEF < 50%) in NYHA classes I-II; Group 3 (n = 23), CD with ventricular systolic dysfunction (LVEF < 50%) in NYHA classes III-IV; Group 4 (n = 22), DCM with ventricular systolic dysfunction (LVEF < 50%) in NYHA classes I-II; and Group 5 (n = 26), DCM with ventricular systolic dysfunction (LVEF < 50%) in NYHA classes III-IV.

Patients were followed for incidences of cardiac death or heart transplant from the time the blood sample was obtained for measurement and analysis of cytokines until end-points were reached or until the follow-up closing date in January 2006. The end-point was defined as either heart transplantation or death. Long-term follow-up of each patient was conducted by medical visit or telephone interview every three months. For those hospitalized during follow-up, the hospital records were reviewed. For all subjects who died, the nearest relative was contacted.

### Plasma sampling and cytokines measurement

Ten milliliters of blood samples were taken from the antecubital vein of subjects and transferred into tubes containing ethylenediamine tetra-acetic acid (EDTA). Immediately after sampling, plasma was separated by centrifugation at 4,000g for 10 min, and frozen at -80°C for further analysis.

Plasma concentrations of twenty-one different human cytokines were measured using the Bio-Plex ProSystem that combined the principle of a sandwich immunoassay with the Luminex fluorescent-bead-based technology (Bio-Rad Laboratories) [22].

### Statistical analysis

The plasma concentrations of all 21 cytokines were expressed as mean ± standard error of the mean (SEM). The associations between concentrations of various cytokines and clinical variables were tested using Mann-Whitney U test. As the data were normally distributed, Pearson’s correlation coefficient (r) was used to analyze the correlation between the investigated cytokines in CD and DCM patient groups and various echocardiographic parameters–they were tested against the null hypothesis that the correlation coefficient is 0. A *p* value < 0.05 was defined as significant. Kaplan-Meier curves were also drawn to compare the survival or necessity for heart transplantation of patients with CD and DCM depending on different concentrations of cytokines. Multivariate analysis was performed using imputing method. By using multivariate analysis, this study tried to investigate whether two or more cytokines together could demonstrate any significant predictive or prognostic potency in patients with CD and in patients with DCM due to idiopathic causes.

## Results

### Patients

Baseline characteristics of patients and control subjects according to the NYHA functional class, ECG, echocardiographic parameters, and medications are given in **Table 1**. The mean duration of follow-up was 36.9 months (range: 13–54 months). At the end of study, the survival status of all patients was known; 30 patients had died and 12 patients had received a heart transplant. Of these who died, 18 patients had CD and 12 patients DCM, while 6 patients each from CD and DCM groups had received a heart transplant.

**Table 1:** Patient characteristics.

Groups	Control		Chagas’ disease		DCM	
		0	NYHA I-II	NYHA III-IV	NYHA I-II	NYHA III-IV
	(n = 25)	(n = 46)	(n = 25)	(n = 23)	(n = 22)	(n = 26)
**Age (years)**	53.9 ± 2.9	52.0 ± 1.5	50.6 ± 2.3	48.9 ± 1.9	49.8 ± 3.6	47.2 ± 2.4
**Sex**						
Male	12	11	10	13	14	16
Female	13	35	15	10	8	10
**SBP (mmHg)**	121.0 ± 2.1	125.7 ± 2.1	110.3 ± 2.2**###**	98.7 ± 2.9***/###	112.8 ± 2.8**#**	95.9 ± 4.6***/###
**DBP (mmHg)**	76.4 ± 0.98	78.9 ± 1.2	73.3 ± 1.3	70.3 ± 1.8**###**	73.9 ± 1.5	70.8 ± 1.8**###**
**HR (beats/min)**	73.6 ± 1.2	69.5 ± 1.3	69.8 ± 2.1	75.0 ± 2.5	72.9 ± 2.0	83.2 ± 2.2**/###
**ECG**						
Normal/Abnormal	25/0	13/33	0/25	0/23	0/22	0/26
Pacemaker	0	4	6	3	1	1
**ECHO**						
LVEF (%)		67.5 ± 1.1	37.8 ± 2.0**###**	24.5 ± 1.5**###**	37.2 ± 2.2**###**	23.4 ± 1.3**###**
LVEF>50%/<50%		46/0	2/23	0/23	0/22	0/26
LVDD (mm)		51.9 ± 1.0	62.7 ± 1.6**###**	70.9 ± 1.3**###**	64.7 ± 2.1**###**	74.9 ± 2.0**###**
LVSD (mm)		32.6 ± 0.90	50.6 ± 1.8**###**	62.1 ± 1.3**###**	52.8 ± 2.1**###**	66.6 ± 2.1**###**
LVEDV (ml)		131.5 ± 6.8	204.1 ± 12.0**#**	303.4 ± 20.2**###**	217.7 ± 16.8**#**	358.4 ± 36.3**###**
LVESV (ml)		41.8 ± 3.0	109.8 ± 10.7**##**	209.5 ± 10.7**###**	137.5 ± 13.5**###**	256.9 ± 28.8**###**
**Pharmacotherapy**						
No medication	25	31	1	-	-	-
ACEI	-	3	22	21	20	15
ß-blocker	-	1	2	-	14	8
ARB	-	-	1	1	2	8
Amiodarone	-	4	5	6	3	2
Diuretic	-	3	17	21	19	25
Digoxin	-	2	11	19	12	22
Spironolactone	-	-	4	14	13	19
Anticoagulant	-	3	2	6	2	8

Data given as mean ± S.E.M.

**p < 0.01

***p < 0.001 *vs*. Control

^**#**^*p* < 0.05

^**##**^*p* < 0.01

^**###**^*p* < 0.001 *vs*. Chagas group 0. ACEI: Angiotensin converting enzyme inhibitor, ARB: Angiotensin receptor blocker, DBP: Diastolic blood pressure, ECG: Electrocardiogram, ECHO: Echocardiography parameters, HR: Heart rate, LVDD: Left ventricular diastolic diameter, LVEDV: Left ventricular end-diastolic volume, LVESV: Left ventricular end-systolic volume, LVSD: Left ventricular systolic diameter, ml: milliliter, mm: millimeter, SBP: Systolic blood pressure.

Systolic blood pressure was significantly altered in patients in the CD group and in patients in the DCM group with advancing HF in comparison with the control group or patients with CD without ventricular systolic dysfunction. **Table 1** also illustrates increased impairment of cardiac function in patients with CD and in patients with DCM with increasing NYHA class.

All patients with ventricular dysfunction received maximal medical therapy according to their functional classes and the treatment guideline recommendations, as shown in **Table 1**. Almost all patients with ventricular dysfunction were given angiotensin converting enzyme inhibitors (ACEI) or angiotensin receptor blockers (ARB); 22 patients with DCM were taking carvedilol, but only 3 patients with CD were receiving beta blocker therapy.

### Data analyses

Plasma concentrations of 21 different cytokines were measured in control subjects, in CD patients, and in DCM patients **(Table 2)**. The CD patients were divided into asymptomatic (0), NYHA classes I-II, and NYHA classes III-IV, while DCM patients were divided into NYHA classes I-II and NYHA classes III-IV.

**Table 2:** Plasma concentrations of cytokines in control, CD, and DCM groups expressed as mean ± standard error of the mean (SEM).

Cytokines	Control		CD 0		CD I-II		CD III-IV		DCM I-II		DCM III-IV	
	Mean	SEM	Mean	SEM	Mean	SEM	Mean	SEM	Mean	SEM	Mean	SEM
**IL-1alpha**	14.63	3.85	15.38	2.89	16.19	4.15	12.71	3.57	13.78	3.82	13.79	5.12
**IL-16**	319.6	28.9	309.7	19.1	322.5	19.2	344.6	30.2	374.9	30.8	354.8	22.3
**IL-18**	139.1	22.2	146.2	21.3	134.4	20.6	171.2	28.6	182.9	31.9	157.5	30.2
**M-CSF**	28.02	5.25	24.71	4.29	28.01	4.86	35.55	6.82	27.22	6.69	26.97	5.45
**MIF**	162.9	19.2	206.1	24.3	189.3	26.3	324.5*	64.5	171.0	21.6	196.9	20.7
**SCF**	195.0	18.5	196.0	16.6	201.5	20.9	223.1	26.9	219.9	26.1	228.4	23.0
**SCGF beta**	24,130	4856	22,943	2,638	26,103	5,226	**46,231#**	9,495	28,944	6,168	**45,903#**	7,705
**TNF**	17.04	3.29	14.88	2.33	15.35	2.87	12.90	2.44	18.48	3.79	14.62	4.15
**TRAIL**	148.0	16.9	152.7	11.3	182.9	24.8	192.3	26.1	170.0	21.8	186.1	28.5
**IL-12p40**	130.6	19.7	171.9	17.2	178.5	27.7	260.3	50.4	172.0	26.5	246.0	41.8
**IL-2R alpha**	100.9	10.3	102.7	7.30	104.6	12.3	123.0	16.0	95.63	7.60	125.1	12.5
**IL-3**	73.29	8.88	82.60	7.43	79.53	8.00	91.00	10.3	84.69	9.15	98.01	9.56
**HGF**	240.2	27.4	242.5	16.9	284.0	36.5	649.0***/**###**	91.4	321.4	30.8	550.5***/###	54.9
**IFN-alpha2**	57.79	4.08	57.73	3.09	58.36	3.91	63.97	5.43	61.84	3.95	69.96	3.48
**beta-NGF**	18.30	4.13	16.28	2.62	19.91	4.38	22.51	4.44	22.44	5.60	26.23	5.87
**LIF**	76.14	4.10	51.46	7.56	53.96	9.15	44.19	9.09	54.69	11.1	56.71	10.8
**CCL27**	1,436	77.0	1,317	70.0	1,519	121	**1,798#**	220	1,504	101	1,480	101
**CXCL1**	45.48	8.95	42.44	6.22	48.10	6.13	56.01	7.87	39.94	4.87	42.47	4.87
**CCL7**	19.74	3.74	17.26	1.46	15.96	2.10	24.72	4.90	15.75	2.85	23.65	3.20
**CXCL9**	808.4	147	1,224	163	1,695	324	2,329***/$ $	412	832.8	95.3	1,307	211
**CXCL12**	138.6	21.3	173.7	18.3	178.8	26.6	214.2	35.4	164.7	24.2	266.5*	30.0

All values are expressed in picograms per milliliter (pg/mL).

*p< 0.05 vs control.

# *p*< 0.05 vs CD 0.

***p< 0.001 vs control.

^###^*p*< 0.001 vs CD 0.

^$ $^*p*< 0.01 vs DCM I-II.

The mean plasma concentrations of these cytokines were then correlated with various echocardiographic parameters such as LVDD, LVSD, LVEDV, LVESV, and LVEF, as shown in **Table 3**. HGF demonstrated a strong correlation with LVEF in CD patients (*p*< 0.001). It also showed a significant correlation with LVSD and LVESV in CD patients (*p*< 0.05). On the other hand, mean plasma CXCL9 concentration was significantly correlated with LVEF in DCM patients (*p*< 0.05), but not in patients with CD. Other cytokines, such as beta nerve growth factor (beta NGF) and monocyte chemo-attractant protein-3 (CCL7; also known as MCP-3) also showed significant correlations with LVEDV and LVEF, respectively, in CD patients (*p*< 0.05).

**Table 3:** Correlation of 21 different cytokines with echocardiographic parameters in CD (NYHA I-IV) and DCM (NYHA I-IV) patients.

CYTOKINES	LVSD		LVDD		LVESV		LVEDV		LVEF	
	CD	DCM	CD	DCM	CD	DCM	CD	DCM	CD	DCM
**IL-1α**	.307	.477	.206	.619	.374	.799	.342	.962	.998	.680
**IL-16**	.890	.769	.896	.739	.789	.948	.152	.581	.520	.830
**IL-18**	.680	.156	.927	.238	.569	.191	.432	.149	.449	.146
**M-CSF**	.207	.364	.319	.343	.415	.795	.261	.914	.278	.734
**MIF**	.106	.851	.103	.894	.344	.545	.978	.615	.231	.405
**SCF**	.459	.232	.552	.308	.753	.364	.469	.701	.662	.304
**SCGF-β**	.292	.367	.636	.460	.286	.613	.595	.968	.101	.292
**TNF**	.830	.515	.752	.520	.946	.552	.446	.664	.911	.655
**TRAIL**	.412	.851	.370	.841	.999	.248	.960	.252	.356	.431
**IL-12p40**	.588	.433	.874	.552	.891	.965	.311	.492	.221	.228
**IL-2Rα**	.356	.476	.428	.628	.700	.820	.286	.670	.414	.366
**IL-3**	.493	.667	.698	.790	.551	.602	.550	.274	.208	.426
**HGF**	.017*	.191	.140	.365	.024*	.071	.398	.166	.0007***	.104
**IFN-α2**	.216	.682	.228	.794	.694	.706	.547	.322	.271	.156
**β-NGF**	.289	.940	.371	.936	.344	.599	.024*	.372	.375	.705
**LIF**	.806	.532	.906	.859	.871	.684	.266	.907	.313	.184
**CCL27**	.560	.906	.873	.962	.848	.405	.335	.226	.391	.748
**CXCL1**	.313	.599	.278	.622	.606	.536	.785	.884	.740	.695
**CCL7**	.063	.640	.156	.859	.147	.159	.643	.156	.028*	.214
**CXCL9**	.394	.486	.644	.656	.509	.389	.885	.471	.276	.033*
**CXCL12**	.488	.094	.673	.163	.612	.342	.394	.163	.271	.085

Echocardiographic parameters include LVSD, LVDD, LVESV, LVEDV, and LVEF.

*p<0.05

***p< 0.001.

**Fig 1:**
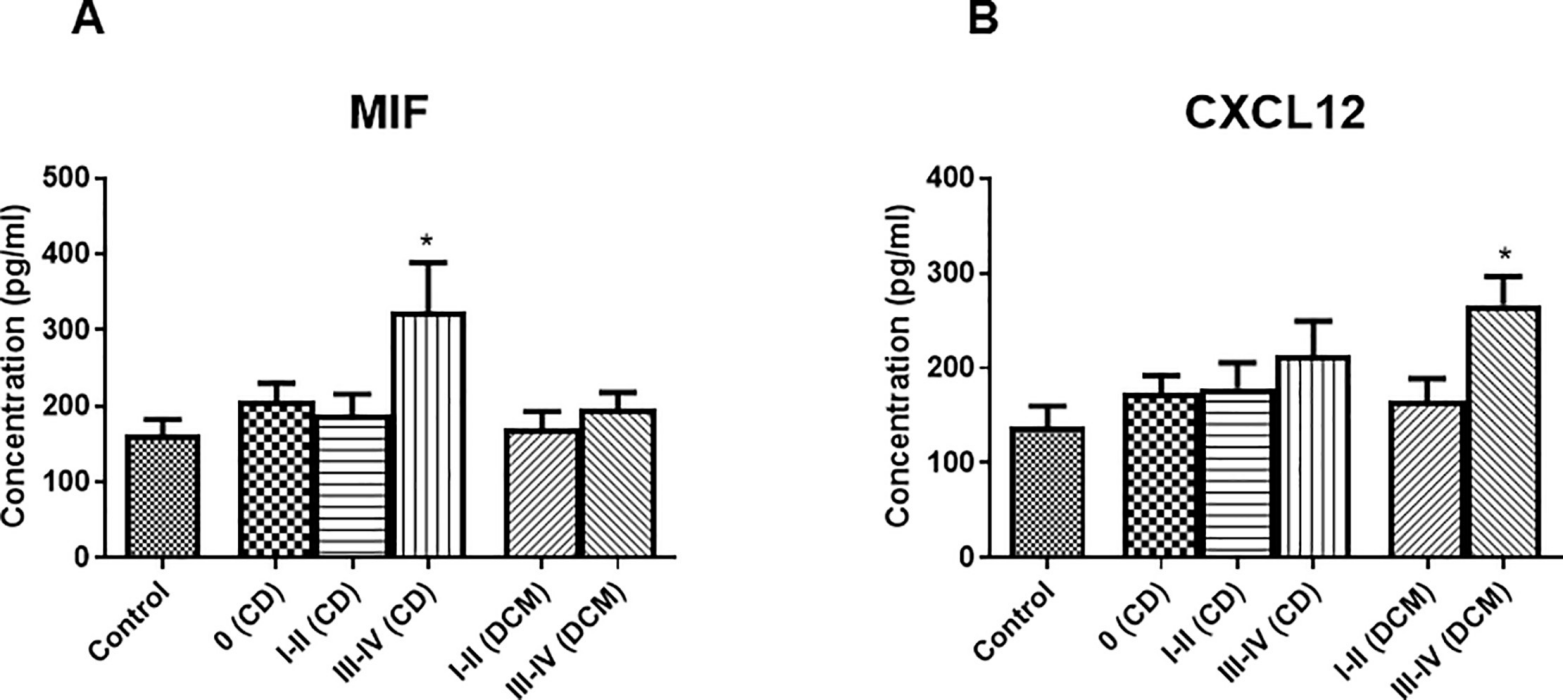
**A.** Plasma concentration of MIF in controls (n = 25); in patients with CD distributed in asymptomatic (0) (n = 46), NYHA classes I-II (n = 24), and NYHA classes III-IV (n = 23); and in patients with DCM divided in NYHA classes I-II (n = 22) and NYHA classes III-IV (n = 26). Data is given as mean ± SEM. * ***p*** < 0.05 vs control. **B.** Plasma concentration of CXCL12 in controls (n = 21); in patients with CD distributed in asymptomatic (0) (n = 44), NYHA classes I-II (n = 25), and NYHA classes III-IV (n = 23); and in patients with DCM divided in NYHA classes I-II (n = 22) and NYHA classes III-IV (n = 26). Data is given as mean ± SEM. * ***p*** < 0.05 vs control.

Mean plasma levels of stem cell growth factor beta (SCGF beta) and hepatocyte growth factor (HGF) were found to be significantly increased in CD and DCM patients with advanced HF (NYHA III-IV) compared to control group [17,18]. While plasma concentrations of macrophage migratory inhibitory factor (MIF) **(Fig 1A)** and CXCL9 were raised significantly only in CD patients with advanced HF and not in DCM patients with advanced HF. **Fig 1A** also demonstrates markedly higher concentration of MIF in CD patients with advanced heart failure in comparison to DCM patients with advanced heart failure (NYHA III-IV) (*p* = 0.0538). In contrast, plasma concentration of stromal derived factor-1alpha (CXCL12; also known as SDF-1alpha) was found to be significantly increased only in DCM patients with advanced HF compared to control group **(Fig 1B)**. The remaining cytokines included in the study showed no significant variation in plasma concentration compared to control in both CD and DCM groups.

**Fig 2:**
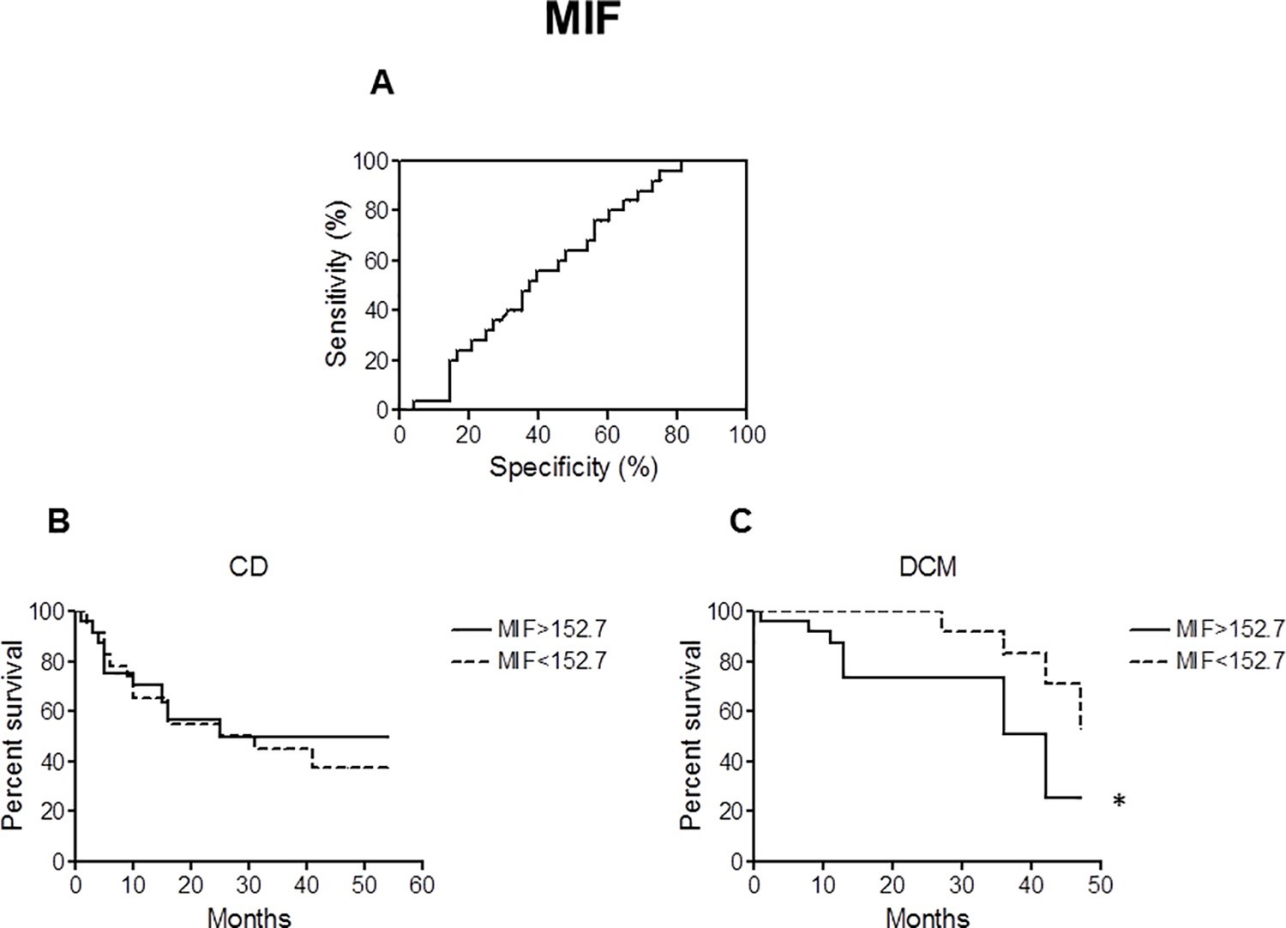
Receiver operating characteristic (ROC) and Kaplan-Meier curves (MIF). **A.** ROC curve was used to define cut-off value for MIF with best sensitivity and specificity based on CD patients in NYHA classes I-IV (Sensitivity: 64.00%; Specificity: 52.08%). The cut-off value was calculated to be 152.7 pg/mL. **B, C.** Kaplan-Meier survival curves were generated to compare percent survival in CD **(B)** and DCM **(C)** patients with MIF higher or lower than cut-off value (cut-off = 152.7 pg/mL); * ***p*** < 0.05.

**Fig 3:**
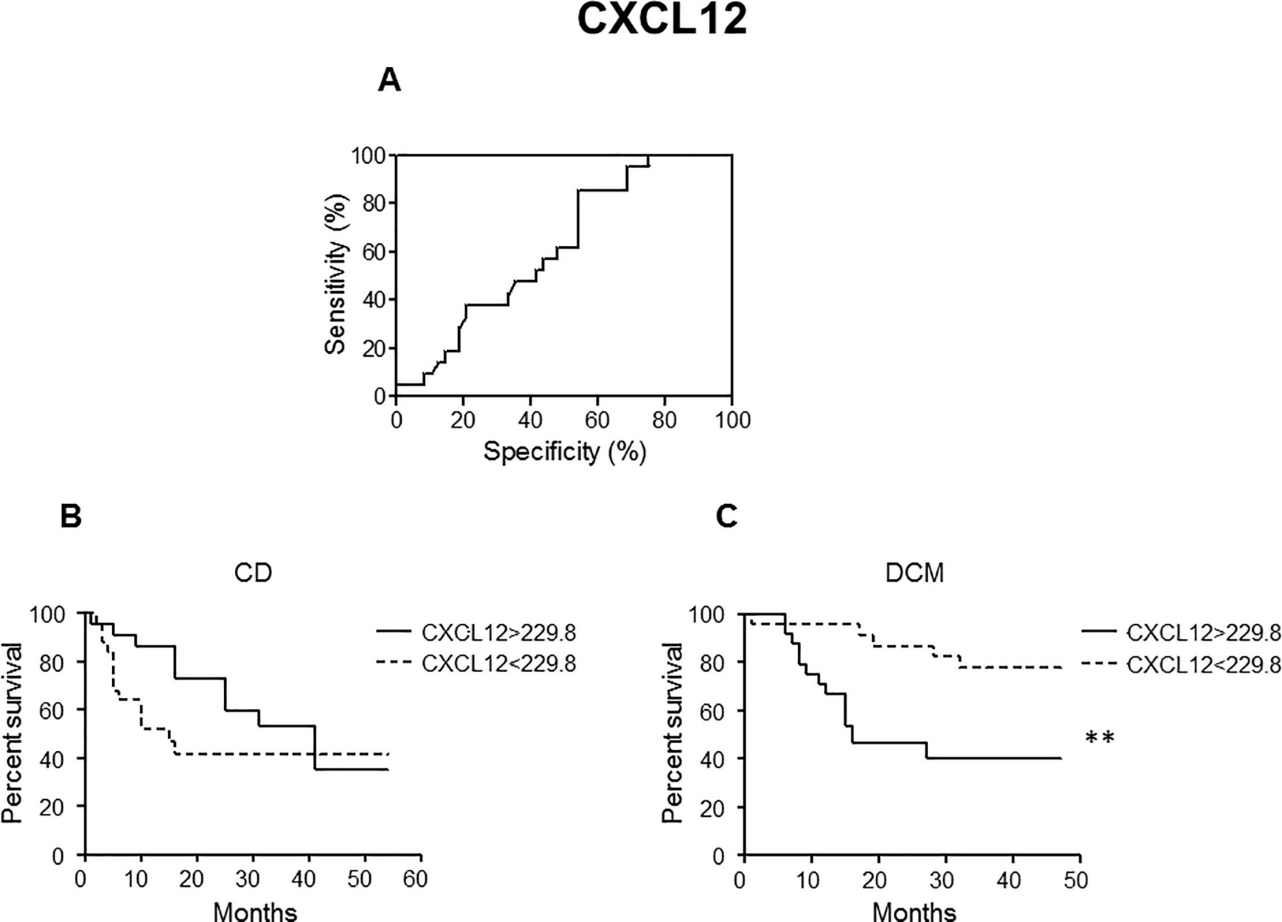
ROC and Kaplan-Meier curves (CXCL12). **A.** ROC curve was used to define cut-off value for CXCL12 with best sensitivity and specificity based on CD patients in NYHA classes I-IV (Sensitivity: 85.71%; Specificity: 45.83%). The cut-off value was calculated to be 229.8 pg/mL. **B, C.** Kaplan-Meier survival curves were generated to compare percent survival in CD **(B)** and DCM **(C)** patients with CXCL12 higher or lower than cut-off value (cut-off = 229.8 pg/mL); ** ***p*** < 0.01. Statistical analysis were carried out by log-rank test.

Receiver operating characteristic (ROC) curves were then generated to calculate cut-off values for plasma MIF **(Fig 2A)** and CXCL12 **(Fig 3A)** concentrations. In order to determine whether the cut-off values for MIF (152.7 pg/mL) and CXCL12 (229.8 pg/mL) have any prognostic potency for mortality and heart transplantation of patients with CD and DCM, Kaplan-Meier curves were drawn using these cut-off values to divide the patients with CD and patients with DCM into two subgroups. While classical statistics failed to identify significant differences or at least a trend in CD patients (NYHA I-IV) for both MIF **(Fig 2B)** and CXCL12 **(Fig 3B)**, they were able to visualize significant predictive value of MIF (*p*< 0.05) and CXCL12 (*p*< 0.01) plasma concentrations for risk in lethality and necessity for heart transplant in DCM patients **(Figs 2C and 3C)**.

Next, multivariate analysis was performed in order to investigate whether two or more cytokines together could demonstrate any significant predictive and prognostic potency in patients with CD and in patients with DCM due to idiopathic causes.

In CD patients, HGF was the only cytokine that was found to be significant univariately. A forward stepwise discriminant analysis selected HGF and growth related oncogene alpha (CXCL1; also known as GRO alpha) as ’the best’ multivariate parameters and was able to separate 70.9% (cross-validated sensitivity/specificity of 31.8%/84.4%) survivors from the deceased. When imputing method was applied, more parameters for the multivariate analysis were allowed (patients with only one of 21 cytokines value missing were usually excluded from the forward stepwise discriminant analysis). In the original data analysis, only 40.5% of patients were considered (N = 45). By imputing, this value was increased to 84.7% of patients (N = 94). Multivariately, the following best combination of cytokine values was selected: HGF and interleukin-12p40 (IL-12p40) with a cross-validated separation of 81.9% (sensitivity/specificity of 92.9%/50.0%), an improvement of 11% in comparison to HGF alone.

In DCM patients, interleukin-16 (IL-16) was significant using univariate analyses. In forward stepwise discriminant analysis, IL-16 and interleukin-2receptor alpha (IL-2R alpha) were selected as ’best’ parameters and was able to correctly classify 66.7% (cross-validated sensitivity/specificity of 74.2%/52.9%) survivors from the deceased. However, in these forward stepwise discriminant analyses, only 32.3% of patients (N = 20) were available. By imputing, it was increased to 77.4% of patients (N = 48). The DCM group now contained almost 80% of complete data. Using this imputing approach, the best multivariate features were CXCL12, stem cell factor (SCF), and CXCL9, cross-validated with a separation of 77.1% (sensitivity/specificity of 76.5%/77.4%), an improvement of 10% in comparison to the forward stepwise discriminant analysis. Statistically, this is very interesting as none of these three cytokine parameters were found to be significant in univariate analyses.

## Discussion

Inflammatory processes mediated by cytokines play a key role in the pathogenesis and progression of CD [23]. This study measured plasma concentrations of 21 different cytokines in control group, in CD patients, and in DCM patients, whereby various of the cytokines have been described for the first time in patients with CD or have never been measured in any kind of cardiac pathology.

It was observed that cytokines were regulated in four major patterns. One group of cytokines showed significant elevation in plasma levels only in CD patients with advanced HF (NYHA III-IV). These included cytokines such as MIF, CCL27 (also known as CTACK), and CXCL9. While, CXCL12 was the only cytokine that demonstrated significant increase in plasma concentration in DCM patients with advanced HF patients but not in CD patients. On the other hand, HGF and SCGF-beta were found to be significantly elevated in both CD and DCM patients with advanced HF. The remaining cytokines included in the study, however, did not show any significant variation in their plasma concentrations in CD or DCM groups.

Owing to the intensity and severity of inflammation in CD, there is a strong possibility that various other cytokines and chemokines apart from the ones that we already investigated are regulated in CD, and could potentially be used, alone or in combination, as diagnostic and prognostic markers in the future. The immunological imbalance between pro- and anti-inflammatory cytokines is largely responsible for the myocardial damage seen in CD [24]. Pro-inflammatory cytokines such as interferon gamma, TNF, and IL-6 are already known to be elevated in serum of CD patients [23,25], while the levels of anti-inflammatory cytokines, IL-10 and IL-17, are reduced with increasing severity of HF in CD patients. Also, cytokines that promote fibrosis and extracellular matrix remodeling might be regulated in CD as cardiomyopathy due to CD is characterized by diffuse, widespread fibrosis within the myocardium [26,27]. Therefore, further studies are required to identify other pro- and anti-inflammatory cytokines and those that induce fibrosis and extracellular matrix remodeling.

There were also some cytokines in this study, such as SCGF beta and CXCL9, whose role in HF from various causes remains to be further clarified. SCGF beta, for instance, is a novel human growth factor for hematopoietic progenitor cells [28,29], that had never been reported in HF patients prior to present study. CXCL9 is a chemokine, which acts as a signaling molecule, regulating the movement of immune cells, directing them to sites of tissue injury and inflammation and modulating their states of activation and effecter cell function [30]. Its expression in heart and circulation can be significantly increased in myocardial infarcted rats [31]. CXCL9 was also previously demonstrated to be up-regulated in patients with Chagasic esophagopathy [32], and it may be a master mediator of myocardial inflammation in patients with CD [33]. Although, SCGF beta and CXCL9 failed to show any prognostic potency in CD and idiopathic DCM patients, the significant increase in plasma levels of both cytokines as seen in advanced HF patients with CD encourages the need for further studies with more patients per specific etiology in order to explore their possible use as diagnostic and/or prognostic markers in CD and idiopathic DCM.

In case of MIF and CXCL12, classical statistics were able to visualize significant predictive value of plasma concentrations of these cytokines for risk in mortality and necessity for heart transplant in DCM patients **(Figs 2C and 3C)**. This is in agreement with recent reports [34,35]. However, no such trend was seen in CD patients (NYHA I-IV) for both MIF and CXCL12 **(Figs 2B and 3B)**. The clear trend in discrimination using a cutoff value in patients with idiopathic DCM but not in patients with CD might implicate that broader inflammation being in part independent by the actual NYHA class in CD might blunt the ability of MIF and CXCL12 to predict mortality and heart transplant.

Multivariate analysis was used in this study in order to investigate whether two or more cytokines together could show any efficacy in diagnosing and prognosticating patients with CD. Most of the cytokines in present study was regulated in the same manner. Cytokines such as MIF, SCGF beta, HGF, CCL27, and CXCL9 only showed significant elevation in plasma levels in CD patients with advanced HF (NYHA III-IV). There were no significant changes in concentrations of cytokines in asymptomatic CD group or in CD patients with mild to moderate HF (NYHA I-II). In DCM group as well, plasma concentrations of SCGF beta and HGF were increased markedly only in patients with advanced HF (NYHA III-IV). Therefore, multivariate analysis was unable to find any combination of two or more cytokines that could help in discriminating CD from idiopathic DCM.

However, multivariate analysis did show some efficacy in prognosticating patients with CD and patients with DCM due to idiopathic causes. In case of CD group, it identified two cytokines by imputing, HGF and IL-12p40, which were able to separate 81.9% of survivors from the deceased (sensitivity/specificity of 92.9%/50.0%). Likewise, in DCM group, CXCL12, SCF, and CXCL9 were selected as best parameters through imputing, and were able to separate 77.1% of survivors from the deceased (sensitivity/specificity of 76.5%/77.4%).

Although, multivariate analysis was unable to find a group of cytokines that could together discriminate CD from DCM due to idiopathic causes, it was, however, able to find a number of cytokines that prognosticated a large number of CD patients and idiopathic DCM patients. In future, further studies need to be done in order to identify cytokines that are regulated differently from one another and could possibly have greater efficacy together in diagnosing and prognosticating patients with CD and DCM.

Considering that HF due to CD is associated with poor prognosis and survival [27,36,37], there is a need to identify those chronically infected, asymptomatic patients with potential to develop HF for early treatment, and distinguish them with DCM. Combination of different spectrum of serum biomarkers may pave the way for such purpose. Our study builds the ground for such an approach qusantifying different cytokines, but it needs the measurement of further cytokines to increase the predictive properties and to generate diagnostic tools. Expanding our approach would help in ensuring that the necessary treatment is provided in time to such patients in order to prevent the onset and progression of HF and thus prevent death. Such biomarkers would also help in prognosis and risk stratification of patients with CD, and in identifying patients who will benefit from heart transplantation.
